# Investigating the relationship between interoceptive accuracy, interoceptive awareness, and emotional susceptibility

**DOI:** 10.3389/fpsyg.2015.01202

**Published:** 2015-08-24

**Authors:** Giuseppe Calì, Ettore Ambrosini, Laura Picconi, Wolf E. Mehling, Giorgia Committeri

**Affiliations:** ^1^Laboratory of Neuropsychology and Cognitive Neuroscience, Department of Neuroscience, Imaging and Clinical Sciences, University G. d'AnnunzioChieti, Italy; ^2^Institute for Advanced Biomedical Technologies, University G. d'AnnunzioChieti, Italy; ^3^Department of Psychological, Health and Territory Sciences, University G. d'AnnunzioChieti, Italy; ^4^Department of Family and Community Medicine, Osher Center for Integrative Medicine, University of California, San FranciscoSan Francisco, CA, USA

**Keywords:** interoception, personality, heartbeat, MAIA, interoceptive awareness, interoceptive accuracy, emotional susceptibility

## Abstract

Interoception, the sense of the physiological condition of the body, provides a basis for subjective feelings and emotions. Anterior insular cortex activity represents the state of the body and varies according to personality traits, such as emotional susceptibility (ES)—the tendency to experience feelings of discomfort and vulnerability when facing emotionally-laden stimuli. The accuracy of perceiving one's own bodily signals, or interoceptive accuracy (IAc), can be assessed with the heartbeat perception task (HPT), which is the experimental measure used by most of the existing research on interoception. However, IAc is only one facet of interoception. Interoceptive awareness (IAw) is the conscious perception of sensations from inside the body, such as heart beat, respiration, satiety, and the autonomic nervous system sensations related to emotions, which create the sense of the physiological condition of the body. We developed an Italian version of the recent self-report Multidimensional Assessment of Interoceptive Awareness (MAIA), tested its psychometric properties (reliability, dimensionality, and construct validity), and examined its relationship to ES, as assessed using the Emotional Susceptibility Scale, in a sample (*n* = 321) of healthy Italian psychology students (293 females, mean age: 20.5 years). In a subgroup of females (*n* = 135), we measured IAc with the HPT. We used a series of correlation/regression analyses to examine the complex interplay between the three constructs. We provide further evidence for a substantial independence of the IAc and IAw measures, confirming previous reports and current theoretical models that differentiate between IAc and IAw. Our analyses elucidate the complex relationship between distinct dimensions of IAw and ES, highlighting the need for continued efforts to shed more light on this topic.

## Introduction

Interoception has been classically conceived of as the sense of the physiological condition specifically of the viscera, as opposed to the five senses (see also Sherrington, 1948). However, subsequent research has led to a redefinition of the classical conception of interoception as the sense of the physiological condition of the entire body (Craig et al., 1996, 2000; Craig, 2002; Saper, 2002). The neurological correlates of interoceptive processes have been well-defined. They convey information essential to the maintenance of an optimal physiological balance in the body—the physiological system's homeostasis—through autonomic, neuroendocrine, and behavioral responses. Interoception has thus been proposed as a core facet of motivational regulation of behavior and cognition (Craig, 2002; Singer et al., 2009). The progressive meta-representation of the physiological condition of the body from posterior to anterior insula (Farb et al., 2013) enables an integration of homeostatic afferent inputs with information from other limbic regions involved in motivation and emotions and from the prefrontal cortex involved in planning (Chikama et al., 1997; Adolphs, 2002; Brooks et al., 2002; Craig, 2002; Olausson et al., 2002). Perception and feedback of interoceptive signals is considered an essential aspect in many theories of emotion (Darwin, 1873; James, 1884; Schachter and Singer, 1962; Damasio, 1994) and has become the subject of research exploring the relationship between interoception and emotional experience.

Interestingly, activity of the anterior insular cortex also appears to be modulated by specific personality traits, such as neuroticism or emotional susceptibility (ES). This latter personality trait, measured by self-report through the Emotional Susceptibility Scale (Caprara et al., 1985), relates to the tendency to experience feelings of discomfort, helplessness, inadequacy, and vulnerability after exposure to emotionally salient stimuli. ES is viewed as reflecting an individual's inclination to experience a state of negative affect and as a tendency to place oneself in a defensive position due to the inability to control excitement, arousal, and reactions in situations—real or imagined—of danger, offense, threat, or attack. Therefore, this personality trait may be of particular relevance for individual differences in reactions to emotional stimuli, particularly to the experienced intensity of affective states, or their arousal dimensions (Bradley and Lang, 1994), which is reflected in the activity of the autonomic nervous system. In an fMRI study, the ES personality trait has been shown to be associated with insular activity (Iaria et al., 2008): participants with high ES scores, as compared to those with low ES scores, showed greater activity within the anterior insula during processing of visual stimuli with emotional content, independent of the stimuli's valence (i.e., positive vs. negative affect). In another study (Ebisch et al., submitted), high ES participants showed greater anterior insular activity in response to neutral gustatory stimuli when interspersed between distasteful and pleasant-tasting affective stimuli. These findings suggest the implication of interoceptive processes in the modulation of basic emotional processing by the ES trait. Although a potential association between ES and interoception has yet to be explored, there is a growing body of evidence supporting the idea that the insula is a key region involved in interoception and emotion (e.g., Zaki et al., 2012; Ernst et al., 2013). Moreover, how interoception is involved in emotional processing has been studied over the last several years (e.g., Wiens, 2005; Craig, 2008; Pollatos and Schandry, 2008; Garfinkel et al., 2014). These studies have suggested that individuals with stronger interoceptive ability report more intense emotions (e.g., Wiens et al., 2000), place a stronger emphasis on the arousal dimension in reporting their emotional experience (Barrett et al., 2004), and show a stronger link between their body reactions to emotional stimuli and their subjective arousal ratings (Dunn et al., 2010). Moreover, interoceptive accuracy has been shown to be positively correlated to measures of central and peripheral processing of emotional stimuli (Pollatos et al., 2005, 2007; Herbert et al., 2007).

However, investigations of the relationship between interoceptive ability and emotional processing have used a variety of measures of interoception, which has triggered recent attempts at more clearly defining the different constructs and measures related to interoception, such as interoceptive accuracy (IAc) and awareness (IAw) (Farb et al., 2015; Garfinkel et al., 2015). Ceunen et al. (2013), for example, defined IAc as the ability to accurately perceive changes in the homeostatic function and IAw as the conscious perception of these body signals, expressing disagreement with the idea that cardiac interoceptive awareness is reflected in and appropriately assessed as the sensorial accuracy in perceiving one's own cardiac signals (e.g., Herbert et al., 2012). Ceunen et al. (2013) proposed that the equivalence between the two concepts probably derives from the “interoceptive sensitivity hypothesis” (Tyrer, 1973; Ehlers and Breuer, 1992), according to which highly anxious individuals and patients with panic disorder—conditions associated with a high symptom awareness level (IAw)—have highly accurate perceptions of anxiety-related bodily sensations (IAc). Ceunen et al. suggested: “IAw should be taken to mean the cognizant, mindful perception of bodily signals […]. Although IAw can be accompanied by an accurate perception of bodily sensations, such accuracy is not necessarily implied” (Ceunen et al., 2013). This suggestion is clearly supported by recent research, which has led to a more differentiated theoretical model of interoception processes (Garfinkel et al., 2015). However, the definition of IAw we are using here, which was developed in focus groups of clinicians and patients and applied in the recently developed Multidimensional Assessment of Interoceptive Awareness (MAIA; Mehling et al., 2012), does not exactly correspond to that provided by Garfinkel and Critchley (2013) and Garfinkel et al. (2015). The latter was operationalized as “a metacognitive measure that quantifies individuals' explicit knowledge of (and confidence in) their interoceptive accuracy” (Garfinkel and Critchley, 2013, p. 233), or “the correspondence between objective IAc and subjective report, i.e., metacognition” (Garfinkel et al., 2015). This pragmatic operationalization of IAw is based on signal-detection theory and reduces the IAw construct to a measure for the extent to which self-reported confidence of perception predicts the accuracy of perception. Our view of the IAw construct, as applied to this study, is also broader and more complex than the conceptualization of interoceptive sensibility, defined as the “self-perceived dispositional tendency to be internally self-focused and interoceptively cognizant” (Garfinkel et al., 2015) and assessed by self-report questionnaires regarding specific bodily sensations or self-rated confidence in one's perceptual ability. We incorporated the earlier, broader definition by Cameron of IAw as awareness of “the afferent information that arises from anywhere and everywhere within the body …[involving] higher mental processes such as emotions, conscious awareness, and behavior” (Cameron, 2001), differentiated further through qualitative analyses from clinically-oriented focus groups (Mehling et al., 2011). By including these attitudinal, appraisal, and self-regulatory aspects of IAw, our conceptualization of IAw extends beyond interoceptive sensibility, which nevertheless remains as one of the dimensions (“Noticing”) in the multidimensional self-report MAIA (Mehling et al., 2012). The pragmatic operationalization of IAw as metacognition is clearly innovative in that it extends beyond self-report, but it appears to strip the construct of some of its psychological, particularly its regulatory, aspects (Bornemann et al., 2014).

The most commonly applied objective measures of interoceptive ability are two different types of Heartbeat Perception Tasks (HPT): the tracking task (Schandry, 1981), in which the perception of cardiac activity is measured by asking the participant to count his/her heartbeats within a defined time interval, and the discrimination task (Whitehead et al., 1977), in which the participant is asked to discriminate between exteroceptive (auditory or visual) and interoceptive (heartbeat) signals. Both methods provide a measure of the individual's interoceptive accuracy (IAc), which in turn has been taken in various earlier studies as an index of one's awareness of one's own bodily sensations, that is, a measure of interoceptive awareness (IAw). Yet early findings showed that IAw, as assessed by self-report measures, does not necessarily predict actual IAc, as assessed experimentally by HPT (e.g., McFarland, 1975). Consequently, in recent years, numerous authors (Wiens, 2005; Mehling et al., 2009; Ceunen et al., 2013; Garfinkel and Critchley, 2013; Bornemann et al., 2014; Farb et al., 2015; Garfinkel et al., 2015) have highlighted the need to discriminate between IAc and IAw and to better define interoception-related constructs. Still, IAc is currently viewed as the central construct underpinning other interoceptive measures (Garfinkel et al., 2015).

In reviewing existing self-report questionnaires on body awareness, which includes both proprioceptive and IAw, and their psychometric properties, Mehling et al. showed that most of the questionnaires are based on a conceptualization of body awareness that considers the attentional focus on bodily symptoms as a maladaptive expression of anxiety, depression, or somatization (Mehling et al., 2009), which may be associated with ES. Instead, a more recent (and alternative) conceptualization of body awareness considers the ability to recognize subtle bodily signals as a controlled state of sustained attention to events that happen within the body here-and-now, which has been integrated into numerous therapeutic approaches geared to improve this ability. An example is mindfulness: the training of a specific style of attention aimed at a state of mind characterized by non-judgmental acceptance and a sense of self that is rooted in experiencing physical sensations in the present moment with equanimity (Thompson and Varela, 2001; Carruthers, 2008; Fogel, 2009; Grossman, 2015). These more recent considerations formed the basis for the development of the MAIA (Mehling et al., 2012; see also Mehling et al., 2009, 2011), a multidimensional self-report instrument designed to measure interoceptive body awareness. It consists of 32 items evaluating eight aspects grouped into five dimensions. The MAIA attempts to overcome the limitations of previous accounts of IAw (described above) by integrating what appear to be the hallmarks of interoceptive body awareness: (1) its multidimensional nature; (2) the distinction between the anxiety-related hypervigilance and the attentional style to bodily sensations in the present moment (which would represent a step away from the assumptions of the interoceptive sensitivity hypothesis); (3) the differential assessment of essential psychological aspects of the perception and evaluation of bodily sensations. To date, the MAIA has been translated into 13 languages (http://www.osher.ucsf.edu/maia/) but, to our knowledge, only two adaptations with adequate validation have been published: the German (Bornemann et al., 2014) and Chilean (Valenzuela Moguillansky and Reyes Reyes, 2015) versions, which both replicated the global structure of the original MAIA.

The first aim of the present work was to provide a preliminary validation of the Italian MAIA, by analyzing the psychometric properties (reliability, dimensionality, and construct validity) of the Italian translation of the MAIA. To this aim, we applied it to a population mainly composed of female psychology undergraduate students.

Our second aim was to investigate the relationship between the multiple facets composing the IAw construct—as assessed by the MAIA—and both the IAc as assessed by the HPT and the ES personality trait. To this aim, we used a series of correlational and regression analyses to examine the complex interplay between the three measures of IAw, IAc, and ES, which are involved in shaping the way individuals experience their “bodily” feelings and emotional states. At the time of the survey (early 2013), it was still an open question whether IAw, as measured by the 8 MAIA scales, would potentially be correlated with IAc or not. Therefore, we aimed at investigating such a relationship with the hypothesis that these constructs are not correlated, supporting the idea that they are distinct and dissociable dimensions. Second, we hypothesized that IAc may be positively correlated with ES, possibly mediated by the arousal component of emotions. Our third exploratory hypothesis was that different scales of the MAIA for the IAw construct would correlate in a complex way with ES, which may add new details to the relationship between interoception, emotion processing, and personality traits.

## Materials and methods

### Participants

A total of 321 participants, aged between 19 and 27 years (*M* = 20.53, *SD* = 0.88), were recruited for the study. Almost all of them were students of the University G. d'Annunzio of Chieti. The majority of the participants (293, representing approximately 91% of the total sample) were female (mean age = 20.49 years, *SD* = 0.85); 28 were male (mean age = 20.96 years, *SD* = 1.10). This disproportion in the participants' gender reflects the gender distribution in the psychology student population from which the experimental sample was mainly recruited. Of this sample, 135 female participants (mean age = 20.40 years, *SD* = 0.72) volunteered to perform the Heartbeat Perception Task (HPT). Participants gave informed consent prior to their inclusion in the study, which was approved by the ethical committee of the University of Chieti and conducted according to the guidelines of the Declaration of Helsinki.

### Instruments and procedures

#### Italian translation of the MAIA

We assessed the multiple aspects of our participants' interoceptive awareness (IAw) by using a new Italian version of the original English-language MAIA (Mehling et al., 2012). Since no Italian version of the MAIA was available, we first systematically translated it. In a first step, three independent forward translations were produced, two by native Italian speakers proficient in English, and one by a native English speaker proficient in Italian. These translators were not familiar with the IAw construct, but have a background in Psychology. The three resulting provisional Italian versions were then compared, item by item, by two of the authors (EA and GCo) and two other researchers from the Laboratory of Neuropsychology and Cognitive Neuroscience of the University of Chieti (Marcello Costantini and Gianluca Finotti), who were familiar with the IAw construct. Following consensus, a provisional Italian version was drafted. Next, an English native bilingual translator, who had a background in Psychology but who was not familiar with the IAw construct, performed the back-translation into English. Finally, differences between the original English version and the back-translation were identified and discussed with the first author of the original MAIA (WM), and further small corrections were applied to improve and finalize the items. The final Italian version was approved by consensus among the same four researchers from the Laboratory of Neuropsychology and Cognitive Neuroscience named above, as well as by the first author of the original MAIA.

The MAIA is composed of 32 items on a 6-points Likert scale, in which the participant has to rate “how often each statement applies to you generally in daily life,” with ordinal responses coded from 0 (“never”) to 5 (“always”). This multidimensional instrument measures IAw on eight scales: (1) Noticing, the awareness of one's body sensations (4 items); (2) Not-distracting, the tendency not to ignore or distract oneself from sensations of pain or discomfort (3 items); (3) Not-worrying, the tendency not to experience emotional distress or worry with sensations of pain or discomfort (3 items); (4) Attention regulation, the ability to sustain and control attention to body sensation (7 items); (5) Emotional awareness, the awareness of the connection between body sensations and emotional states (5 items); (6) Self-regulation, the ability to regulate psychological distress by attention to body sensations (4 items); (7) Body listening, the tendency to actively listen to the body for insight (3 items); and (8) Trusting: the experience of one's body as safe and trustworthy (3 items). The score for each scale is calculated by averaging the scores of its individual items, and thus can vary in the 0–5 range.

#### Emotional susceptibility scale

We assessed the participants' ES by using the Italian version of the Emotional Susceptibility Scale, a self-administered, unidimensional instrument developed by Caprara et al. (1985; Caprara et al., 1983) to measure the participants' “tendency to experience feelings of discomfort, inadequacy, and vulnerability” after the exposure to emotionally salient stimuli. This instrument is composed of 40 items on a 6-points Likert scale, in which the participant has to rate “how true” each statement is for him/her, with ordinal responses coded from 1 (“completely false for me”) to 6 (“completely true for me”). The ES scale includes 10 control items that do not contribute to the final score, which is obtained by summing the scores of the 30 non-control items.

#### Heartbeat perception task

From our 321 participants, we recruited volunteers to perform the HPT (*n* = 135) in the laboratory. After the participants had arrived, they were informed about the general procedure and the measures. The participants were seated in a comfortable chair in a sound-attenuated room. The HPT procedure was performed according to the mental tracking method proposed by Schandry (1981), which was modified by adding a fourth time interval (Pollatos et al., 2008). The four time intervals were 25, 35, 45, and 100 s and were presented in random order. During each time interval, participants were asked to count and keep track of their heartbeats by focusing on their heart activity and bodily feelings. The participants were explicitly instructed not to take their own pulse in any way or to try any other physical manipulations; they were also discouraged from simply tracking the number of seconds of each time interval and using that information to estimate the number of heartbeats. After each interval, the participants were asked to verbally report the count or estimated number of heartbeats, which was manually recorded by the experimenter.

Participants were distributed in three groups. In the first two groups, the actual number of the participant's heartbeats was recorded by one of the authors (GCa) and a trained assistant by means of either tactile radial arterial palpation at the wrist or direct chest auscultation through a stethoscope. The experimenters kept track of time using a digital stopwatch. The beginning and end of each time interval was indicated to the participants by the acoustic signal emitted by the stopwatch. In the tactile arterial palpation procedure, particular care was taken by the experimenters to avoid applying excessive pressure to the participants' wrist skin, which could have made it easier for the participants to perceive their heartbeats. In the third group, the participant's heartbeats were recorded by another author (EA) and a trained assistant using a three-lead electrocardiogram: three Ag/AgCl pre-gelled electrodes (ADInstruments, UK) were placed on the participant's chest in an Einthoven's triangle configuration to record the electrocardiogram (sampling rate: 1 KHz; Powerlab, ADInstruments, UK); the heartbeats were identified by the R peaks using the BioSig package (Vidaurre et al., 2011) for Matlab (MathWorks, Natick, MA). The beginning and the end of each time interval were indicated to the participant through acoustic signals presented via headphones. The entire procedure was controlled by a computer running the Matlab Psychophysics Toolbox (Brainard, 1997). The participants of this third group were part of a larger study investigating the physiological responses to affective stimuli.

#### General procedure

Participants were asked to complete the MAIA and the ES scale. Both scales were administered in paper-and-pencil versions in classroom settings during psychology courses or, for a small number of participants recruited from campus facilities, in the laboratory setting. Data collection was conducted by the first author (GCa) and trained assistants. The purpose of the study was briefly explained to the participants, who were informed that they were free to respond (but asked to respond truthfully), and that their collaboration would potentially be requested again at a later stage of the study. The HPT was performed in a separate session as detailed in Section Heartbeat Perception Task.

### Data analysis

Missing values for items of the MAIA and ES scale were imputed using the series mean method, that is, by replacing them with the mean of all participants' values for the same item. For the MAIA, there were on average 0.97 missing values (*SD* = 2.63) for each item, whereas the missing values were, on average, 1.18 (*SD* = 1.20) for each item of the ES scale (out of the total of 321 responses per item). The scores of the eight scales of the MAIA were computed by averaging the values of the items of each scale, according to the final factorial structure we obtained (see Section Psychometric Properties and Factorial Structure of the Italian MAIA). The ES score was computed for each participant by averaging the score of the 30 non-control items of the ES scale, thus obtaining a value ranging from 1 to 6.

The participants' performance in perceiving their heartbeats during each time interval of the HPT was calculated as a relative error score, that is, the absolute difference between reported and actual number of heartbeats divided by the actual number of heartbeats. Next, in line with standard practice (e.g., Koch and Pollatos, 2014), the participants' interoceptive accuracy (IAc) score was computed according to the formula: IAc = 1/4 Σ [1 − (|recorded heartbeats − counted heartbeats|/recorded heartbeats)]. We then tested for differences in IAc scores between the three groups of participants (see Section Heartbeat Perception Task). We carried out pairwise comparisons with two-tailed independent-samples *t*-test and the corresponding Bayesian *t*-test (Rouder et al., 2009) for accepting the null hypothesis of no differences between groups.

We first investigated the preliminary psychometric properties and the dimensionality of the Italian version of the MAIA, in order to evaluate whether the factor structure of the original version would replicate in the Italian version. To this aim, we carried out an exploratory factor analysis (EFA) and an analysis of covariances within the framework of confirmatory factor analysis (CFA), and assessed the reliability of the MAIA scales. The hypothesized model was estimated via maximum likelihood (ML). For the evaluation of covariance structure models we used the chi-square goodness of fit supplemented by the comparative fit index (CFI), the root-mean-square error of approximation (RMSEA) and the standardized root-mean-square residual (SRMR). The CFI (Bentler, 1990) assesses the reduction in misfit of a population target model relative to a population baseline model in which no structure is specified (i.e., all correlations among variables are equal to zero). Values of at least 0.90 are considered adequate for good models (Bentler, 1990). The RMSEA is a measure of the discrepancy of the variance covariance matrix of fitted model from the starting variance covariance matrix per degree of freedom. Values lower than 0.05 reflect a small error of approximation and values between 0.05 and 0.08 reflect an acceptable error of approximation. Values greater than 0.10 constitute poor model fit (Browne and Cudeck, 1993). The SRMR is an absolute index of the discrepancy between reproduced and observed correlations. Hu and Bentler (1998) suggested a cutoff criterion of 0.08, with higher values indicating poorer fit to the empirical data and values lower than 0.05 indicating an excellent fit. Finally, the Cronbach's alpha coefficient and corrected item-scale correlations were used to assess the reliability of the scales.

Next, we explored the complex interplay between the three constructs of ES, IAw, and IAc. We first calculated the Pearson correlations between each pair of variables, including ES score, scores of the eight scales of MAIA (as derived from the factorial analyses, see Section Psychometric Properties and Factorial Structure of the Italian MAIA)^1^, and IAc score, with pairwise deletion of cases with missing data. For a more precise investigation of the actual relationship between these measures, partial correlations were calculated between each pair of variables, controlling for all the other variables. We carried out multiple regression analyses to investigate in more detail the relationship between the participants' ES scores (the dependent variable) and the eight MAIA scores and the IAc score (continuous predictors), as well as all two-way interactions. The model that best explained the ES scores, with the appropriate number of predictors, was selected by the method of the best subset as implemented in Statistica (StatSoft). In brief, all possible regression models with up to a defined number (subset) of predictors were evaluated; among those with the same number of predictors, the model with the highest percentage of explained variance (*R*^2^) was provisionally selected as best explaining the ES scores. This procedure identifies the best model for any number of predictors (Neter et al., 1989; as cited in StatSoft Inc, 2013). Finally, models with different numbers of predictors were compared by testing for the significance of the *R*^2^ difference taking into account the tolerance index, a measure of multicollinearity among the predictors included in the model. Statistical analyses were conducted through Statistical Software for Social Science (SPSS), Statistica (StatSoft), and Structural Equation Modeling (EQS).

## Results

We first describe the preliminary psychometric properties and the dimensionality of the Italian MAIA and compare them with those of both the original version and the two other published German (Bornemann et al., 2014) and Chilean (Valenzuela Moguillansky and Reyes Reyes, 2015) translations.

### Psychometric properties and factorial structure of the Italian MAIA

The appropriateness of the factor analysis as a model for analyzing the data was supported by the Bartlett's test of sphericity (χ^2^ = 3129.50*p* < 0.001) and the Kaiser-Meyer-Olkin measure of sampling adequacy (0.822). Assessment of distributions showed that all but one item had skewness and kurtosis values in the (−1, 1) range (item 4 had a skewness value of −1.054, see Table 1).

**Table 1:** **Univariate descriptive item statistics (*n* = 321)**.

**Item**	**Mean**	**SD**	**Skewness**	**Kurtosis**
Item1	2.47	1.23	−0.01	−0.43
Item2	2.94	1.30	−0.34	−0.46
Item3	3.09	1.20	−0.45	−0.28
Item4	3.69	1.24	−0.05	0.72
Item5	2.74	1.36	−0.12	−0.76
Item6	2.79	1.21	0.05	−0.71
Item7	2.43	1.37	0.13	−0.67
Item8	2.32	1.26	0.13	−0.78
Item9	2.70	1.15	−0.02	−0.56
Item10	2.19	1.20	0.10	−0.68
Item11	2.57	1.27	−0.26	−0.58
Item12	2.83	1.13	−0.26	−0.27
Item13	2.77	1.32	−0.32	−0.56
Item14	2.48	1.12	−0.10	−0.31
Item15	2.68	1.02	−0.27	0.07
Item16	2.63	1.16	−0.04	−0.45
Item17	3.01	1.01	−0.03	0.11
Item18	3.00	1.27	−0.40	−0.54
Item19	2.79	1.28	−0.13	−0.57
Item20	3.41	1.14	−0.67	0.14
Item21	3.74	1.16	−0.92	0.44
Item22	3.57	1.16	−0.93	0.58
Item23	2.60	1.19	−0.09	−0.51
Item24	2.44	1.15	−0.03	−0.47
Item25	3.02	1.21	−0.47	−0.22
Item26	2.45	1.21	0.02	−0.56
Item27	2.95	1.08	−0.38	−0.19
Item28	1.98	1.17	0.32	−0.34
Item29	2.02	1.21	0.29	−0.43
Item30	2.80	1.39	−0.19	−0.70
Item31	2.88	1.32	−0.14	−0.56
Item32	3.35	1.16	−0.52	−0.05

For the EFA, we extracted factors using the principal axis factoring method and factor loadings higher than ± 0.30 were considered as building criteria for the EFA model. In order to determine the number of factors to retain, we examined the eigenvalues (Cattell and Vogelmann, 1977) (extraction criterion: eigenvalue > 1; varimax rotation). After the successive deletion of three items (7, 10, and 12) that did not load significantly on the theoretical factor, and after the interpretation of the factor scores, the eight factors solution, accounting for the 47.74% of the total variance, was also corroborated by an inspection of the scree-plot of eigenvalues (the first 10 eigenvalues were 6.566, 2.542, 1.942, 1.679, 1.520, 1.283, 1.170, 1.089, 0.931, and 0.883). This eight factors solution clusters the remaining items as in the original version, with the exception of items 19 (“When something is wrong in my life, I can feel it in my body.”), which loaded on both its original factor Emotional Awareness (0.469) and on Body Listening (0.377) (for a similar result, see Bornemann et al., 2014), and 4 (“I notice changes in my breathing, such as whether it slows down or speeds up.”), which loaded on a different factor (Emotional Awareness: 0.387) than its original factor (Noticing: 0.147). We then carried out a CFA, which showed that the eight factors solution obtained with the EFA provided an adequate fit to the data [χ^2(349)^ = 408.99; *p* = 0.015; RMSEA = 0.023 (90% CI = 0.011, 0.032); SRMR = 0.057, CFI = 0.974].

Table 2 shows mean values, standard deviation, average item-scale correlations, and Cronbach's alphas of the Italian MAIA. The Cronbach's alpha values varied between 0.53 and 0.80. Compared to the alpha values of the original MAIA (Mehling et al., 2012) by means of the Feldt's test for independent samples (Feldt, 1986), the alpha values of the Italian MAIA were significantly lower than in the original MAIA for five scales (Attention regulation, Self-regulation, Body listening, Not-worrying, and Not-distracting), and not statistically different for the remaining three scales. Similar results were obtained when comparing the alpha values of the Italian MAIA with those of both the German and Chilean versions of the MAIA. Notwithstanding these differences, it should be noted that our Cronbach's alpha values were highly correlated to those of the English, German, and Chilean versions (*r* = 0.855, 0.973, and 0.928; all *p*s ≤ 0.007). Moreover, the pattern of values we observed was very similar to that of the other MAIA versions, with the Noticing, Not-worrying, and Not-distracting scales consistently showing the lower Cronbach's alpha values among the scales (all *p*s ≤ 0.0001, Feldt's test for dependent samples; Feldt et al., 1987).

**Table 2:** **Descriptive statistics, reliability indices, and interscale/inter-construct correlations for IAw, ES, and IAc**.

	**Measures**	**Mean**	**SD**	**Cronbach's alpha**	**Item-scale correlation**	**No. of items**	**Interscale/Inter-construct Pearson's correlations^a^**									
	***IAw* (MAIA, *n* = 321)**						1	2	3	4	5	6	7	8	9	10
1	Noticing	2.84	0.97	0.68	0.495	3		0.704	0.278	**<0.001**	**<0.001**	**<0.001**	**<0.001**	**<0.001**	0.316	0.543
2	Not-distracting	2.77	1.06	0.53	0.361	2	0.021		0.107	0.087	0.599	0.887	0.910	0.967	0.695	0.495
3	Not-worrying	2.51	1.01	0.59	0.418	2	−0.061	−0.090		0.344	**<0.001**	0.171	0.214	0.959	**<0.001**	0.352
4	Attention regulation	2.69	0.77	0.75	0.495	6	**0.329**	−0.096	0.053		**<0.001**	**<0.001**	**<0.001**	**<0.001**	**<0.001**	**0.020**
5	Emotional awareness	3.37	0.85	0.79	0.550	6	**0.335**	0.029	−**0.196**	**0.315**		**<0.001**	**<0.001**	**0.002**	**0.001**	0.534
6	Self-regulation	2.63	0.90	0.75	0.540	4	**0.310**	−0.008	−0.076	**0.361**	**0.428**		**<0.001**	**<0.001**	0.103	0.713
7	Body listening	2.32	0.94	0.74	0.569	3	**0.364**	0.006	−0.069	**0.327**	**0.429**	**0.571**		**<0.001**	0.299	0.974
8	Trusting	3.01	1.09	0.80	0.653	3	**0.123**	0.002	0.003	**0.375**	**0.170**	**0.414**	**0.281**		**<0.001**	0.338
9	*ES* (*n* = 321)	4.02	0.92	0.90	0.508	30	0.056	−0.022	−**0.359**	−**0.244**	**0.187**	−0.091	0.058	−**0.331**		0.412
10	*IAc* (HPT, *n* = 135)	0.47	0.19	0.89	0.852	4	−0.053	−0.059	0.081	**0.200**	−0.054	−0.032	−0.003	0.083	−0.071	

a *Values of the Pearson's correlation coefficients (r) are shown in the lower triangle, and the corresponding p-values are shown in the upper triangle. Significant correlations are indicated in bold*.

Table 2 also shows the Pearson's interscale correlations. The results revealed a complex pattern of intercorrelations between the scores of the eight scales of MAIA, with a high percentage of significant correlations (16 out of 28 possible pairs, representing approximately 57%, with α = 0.05). The only two scales that seem to be relatively independent from the other scales were Not-worrying and Not-distracting, which composed the dimension “Emotional Reactions and Attentional Response to a Sensation” in the original MAIA (Mehling et al., 2012). Again, this pattern of results was consistent with that found in the other available versions of the MAIA. In fact, the Pearson's correlations between the interscale correlation matrices of the Italian MAIA, on the one side, and those of the English, German, and Chilean MAIA, on the other side, were very high (respectively, 0.896, 0.925, and 0.925; all *p*s < 0.001), highlighting a high second-order isomorphism (Shepard and Chipman, 1970) of the correlational structure of the MAIA across these three languages and cultures.

### Heartbeat perception task

There were no significant differences in IAc scores between the three groups of participants (see Section Heartbeat Perception Task), suggesting that the method of measurement of the heartbeats (ECG, stethoscope, wrist palpation) did not influence the participants' IAc. Mean IAc scores in the three groups were 0.503 (*SD* = 0.191), 0.542 (*SD* = 0.182), and 0.540 (*SD* = 0.185), respectively. These were not different in pairwise comparisons by *t*-tests [ECG vs. stethoscope: *t*_(90)_ = 0.987, *p* = 0.327; ECG vs. wrist palpation: *t*_(90)_ = 0.721, *p* = 0.473; stethoscope vs. wrist palpation: *t*_(84)_ = 0.939, *p* = 0.350]. More importantly, Bayesian *t*-tests (Rouder et al., 2009) provided support for the null hypothesis across the three methods with JZS Bayes factors of 3.96, 4.90, and 4.02, respectively, for the same pairwise comparisons. Therefore, we felt justified in pooling the data across heartbeat recording methods for our subsequent analyses.

### Inter-constructs relationships

Table 2 shows the Pearson's correlations between the eight scales of the Italian MAIA and both the ES and IAc scores of the participants. The analysis revealed significant correlations between the ES scale and four of the eight scales of the MAIA: Not-worrying, Attention regulation, Emotional awareness, and Trusting (respectively, *r* = −0.359, −0.244, 0.187, and −0.331; all *p*s ≤ 0.001; *n* = 321). The IAc score was weakly but statistically significantly correlated with only one MAIA scale: Attention regulation (*r* = 0.200, *p* = 0.020, *n* = 135).

Given the complex pattern of intercorrelations among the MAIA scales, we also calculated partial correlations between each pair of variables, controlling for all other variables. This analysis essentially confirms the results of the previous one and revealed weak but statistically significant partial correlations (*r*_partial_) between the ES scale and the same four MAIA scales that emerged from the regular correlation analysis (respectively, *r*_partial_ = −0.339, −0.222, 0.212, −0.289; all *p*s ≤ 0.001; *n* = 321), with an additional significant partial correlation for the Body Listening scale (*r*_partial_ = 0.128, *p* = 0.023; Table 3). Moreover, the weak but statistically significant correlation between the IAc score and the MAIA Attention Regulation scale was confirmed by the partial correlation analysis (*r*_partial_ = 0.226, *p* = 0.011, *n* = 135).

**Table 3:** **Partial correlations between IAw scales, ES, and IAc**.

	**Measures**	**Interscale/Inter-construct partial correlations^a^**									
	*IAw* (MAIA, *n* = 321)	1	2	3	4	5	6	7	8	9	10
1	Noticing		0.424	0.970	**<0.001**	**0.022**	0.189	**0.003**	0.350	0.271	0.236
2	Not-distracting	0.045		0.076	**0.021**	0.364	0.658	0.713	0.846	0.094	0.939
3	Not-worrying	0.002	−0.100		0.624	0.075	0.351	0.403	0.095	**<0.001**	0.628
4	Attention regulation	**0.217**	−**0.130**	0.028		**0.001**	0.329	0.112	**0.001**	**<0.001**	**0.011**
5	Emotional awareness	**0.129**	0.051	−0.100	**0.194**		**<0.001**	**0.005**	0.868	**<0.001**	0.644
6	Self regulation	0.074	−0.025	−0.053	0.055	**0.214**		**<0.001**	**<0.001**	0.093	0.487
7	Body listening	**0.165**	0.021	0.047	0.090	**0.157**	**0.403**		0.159	**0.023**	0.685
8	Trusting	−0.053	0.011	−0.094	**0.194**	0.009	**0.242**	0.079		**<0.001**	0.583
9	*ES* (*n* = 321)	0.062	−0.095	−**0.339**	−**0.222**	**0.212**	−0.095	**0.128**	−**0.289**		0.710
10	*IAc* (HPT, *n* = 135)	−0.106	−0.007	0.043	**0.226**	−0.041	−0.062	0.036	0.049	0.033	

a *Partial correlations are shown in the lower triangle with corresponding p-values in the upper triangle. Significant correlations are indicated in bold*.

Since the correlational analyses showed that the IAc score was not reliably related to the ES score, and since a first multiple regression analyses showed that IAc did not reliably predict ES score (neither individually nor in interaction with other predictors), we chose to exclude it from the list of continuous predictors for the multiple regression analysis, in order to have a larger sample of valid cases (*n* = 321, the participants who completed both the MAIA and ES scales, instead of *n* = 135 for the subsample of participants who also performed the HPT) and, thus, increase the statistical power. The subsequent multiple regression analyses, performed with the best subset method as detailed in the Data Analysis Section, revealed a model that included three predictors as the best model to explain the participants' ES scores. This model included, ordered by the percentage of explained variance, the interaction between Attention regulation and Trusting [β = −0.413, *SE* = 0.049, *t*_(317)_ = 8.142, *p* < 0.001], Not-worrying [β = −0.295, *SE* = 0.048, *t*_(317)_ = 6.123, *p* < 0.001], and Emotional awareness (β = 0.241, *SE* = 0.050, *t*_(317)_ = 4.829, *p* < 0.001) as predictors, explaining 29.87% of the variance [*F*_(3, 317)_ = 45.01, *p* < 0.001], with good tolerance values (≥0.883). Therefore, participants with higher ES scores reported more emotional distress or worry with sensations of pain or discomfort (Not-worrying), higher awareness of the connection between body sensations and emotional states (Emotional awareness), and were either less able to sustain and control attention to body sensation (Attention regulation) or less prone to experience their own body as a safe and trustworthy place (Trusting). In particular, the interaction between Attention regulation and Trusting revealed that the participants who had a score near zero in at least one of these scales also had higher ES scores, that is, were more prone to experience feelings of discomfort, helplessness, inadequacy, and vulnerability due to the inability to control their reactions in negative situations (either real or imagined), irrespective of their score in the other scale; conversely, only participants who showed high scores in both these scales reported to have low ES. It is important here to note that the significant effects revealed in this multiple correlation analysis concern the same variables for which we found significant regular and partial correlations with the ES score, thus highlighting the stability of these findings but, at the same time, revealing a more complex pattern of relationships between the ES and the multiple facets of the IAw, as evaluated by the MAIA.

## Discussion

In the present study, we aimed to test the psychometric properties of the new Italian translation of the MAIA and explore the complex interplay between three constructs that contribute in shaping how we experience our “bodily” feelings and emotional states, that is: interoceptive accuracy (IAc), interoceptive awareness (IAw), and emotional susceptibility (ES). First, analyses of the preliminary psychometric properties and the factorial structure of the Italian version of the MAIA revealed that it has acceptable reliability and a dimensionality that is comparable to that of the other available MAIA versions (Mehling et al., 2012; Bornemann et al., 2014; Valenzuela Moguillansky and Reyes Reyes, 2015). However, this validation is limited, and the present data should be considered as a first, preliminary validation of the MAIA in the Italian population, as we tested it in a sample of 321 mostly female undergraduate Psychology students that is hardly representative of the Italian population. Therefore, further examinations on a more representative sample are needed.

Second, our analyses elucidate the complex relationship between distinct dimensions of IAw and ES and add new data to the discriminatory and convergent validity of the MAIA scales. Our results from correlational analyses revealed that the participants' scores on four of the eight MAIA scales, namely Not-worrying, Emotional awareness, Attention regulation, and Trusting were reliably correlated with the participants' ES. Multiple regression analyses further elucidated the specific contribution of each MAIA scale in explaining the variability in the participants' ES. These analyses revealed that, while both the Not-worrying and Emotional awareness scales were independent significant predictors of the participants' ES scores, the Attention regulation and Trusting scales interacted in explaining the (larger portion of the) variability in the participants' ES scores. Our results thus showed that two specific aspects of IAw independently contributed in explaining the variability in participants' ES. First, Not-worrying was significantly and negatively related to ES: participants who reported to be more prone to experience emotional distress or worry in response to negative body sensations (i.e., with a score near zero in the Not-worrying scale) also reported to be more prone to experience discomfort, vulnerability, and inadequacy in controlling their excitement, arousal, and reactions in situations of danger, offense, or attack (Caprara et al., 1983), as evidenced by high ES scores. This result suggests a tight link between these two constructs, which makes sense, as both are related to measures of trait anxiety (see Caprara et al., 1983; Mehling et al., 2012), and situations of danger, threat, offense, or attack all lead to the activation of the sympathetic nervous system, with subsequent strong bodily signals of alarm. As shown by Mehling et al. (2012), the MAIA Not-worrying scale was related to the Physical Concern subscale of the Anxiety Sensitivity Index (Zinbarg et al., 1997), which assesses the proneness or enduring tendency to worry when experiencing bodily sensations of discomfort, such as quickened respiration or heartbeat and chest constriction. These sensations can be triggered by “emotionally salient stimuli” and the “situations of danger, offense, or attack” that contribute to the definition of the ES construct and, therefore, are all phenomena that individuals with high ES experience as a state of negative affect. Thus, to not worry would mean to accept and tolerate these negative body signals, to have a mindful, nonjudgmental acceptance in experiencing the physical sensations in the present moment (Mehling et al., 2012), and not to “burden oneself with them,” while experiencing feelings of helplessness, inadequacy, and vulnerability (Caprara et al., 1985).

Next, although the Emotional awareness scale was significantly related to the participants' ES, those who reported to have high awareness of the connection between their emotional states and body sensations also had high ES. At first glance, this finding may seem counterintuitive: one could expect to find the opposite relation between the Emotional awareness and the ES. However, a recent study provides findings in line with our results. Indeed, Lichev et al. (2015) found that individuals with high emotional awareness showed stronger (implicit) affective reactivity. Moreover, the results of an analysis conducted by Mehling et al. (2012) may provide some insights to help us better understand how the multiple aspects of the IAw relate to anxiety. The authors showed that Emotional awareness was the only scale that showed a specific—even if marginally significant—positive relation with the trait anxiety measure, despite its negative association with anxiety measures in a simple correlational analysis. Follow-up regression analyses showed that a possible cause of this inversion of the association between anxiety and Emotional awareness was the portion of shared variance between the latter measure and another MAIA measure, Self-regulation. In other words, even if Emotional awareness was negatively related to anxiety when taken alone, this relation became positive after having removed the portion of variance shared with Self-regulation. This suggests that the Emotional awareness scale would assess distinct aspects of this construct that may be negatively and positively related to anxiety (respectively, those shared with the Self-regulation scale and those that are specific to the Emotional awareness). This interpretation led the authors to conclude that “mere awareness of how body sensations correspond to emotional states [i.e., the Emotional awareness], without the ability to use awareness of those sensations to reduce distress [i.e., the Self-regulation], could increase anxiety” (Mehling et al., 2012). Therefore, it is possible that the positive relationship we found between Emotional awareness and ES was mostly driven by the same specific aspects that Mehling and colleagues found to be related to an increased trait anxiety, which in turn is strongly related to ES (Caprara et al., 1983). Albeit speculative, this possible explanation highlights the need to conduct further studies and intensify the efforts to clarify how specific aspects of body awareness affect anxiety.

Finally, and more importantly, the multiple regression analysis showed an even stronger relationship between the participants' ES and the interaction between Attention regulation and Trusting. This result is of particular interest, as it reveals a pattern of relationships between the ES and these facets of the IAw that is more complex than it would at first appear by relying on the correlational analyses. The significant interaction between Attention regulation and Trusting reveals their non-additive effect in explaining the ES variability: participants who had a score near zero in either the Attention regulation or the Trusting scale also had higher ES scores, that is, reported to be highly prone to experiencing feelings of discomfort, helplessness, inadequacy, and vulnerability due to the inability to control their reactions in negative situations, irrespective of their score in the other scale; on the contrary, only participants who showed high scores in both these scales reported to have a low ES. This result indicates that, when taken alone, neither of these two abilities is enough to protect oneself against the negative feelings implied by a high ES score. In other words, the inclination to experience the body as a safe and trustworthy place (i.e., a high Trusting score) also needs the concomitant ability to actively direct and maintain the attention on the body signals (i.e., a high Attention regulation score) to allow one to control feelings and reactivity in negative situations. In line with this result, different authors considered the skills in attention regulation as a precondition for the capacity to be nonreactive and tolerant of body sensations, which are key elements of a more general mindfulness approach to experience (e.g., Shapiro et al., 2006; Mehling et al., 2009, 2012; Hölzel et al., 2011). Our data emphasize that focusing attention on body signals can be maladaptive if one does not “feel at home” in his/her own body. Therefore, good attention regulation skills (as assessed by the MAIA) should be considered as just one necessary—but not sufficient—precondition of a beneficial mind-body relation and emotional stability, even when it is viewed as related to a “positive” mode of mind, or mindful presence (Mehling et al., 2009), (i.e., “focusing attention directly on immediately experienced feelings,” instead of “[having an] abstract ruminative self-focus,” Mehling et al., 2012; see also Watkins and Teasdale, 2004; Shapiro et al., 2006).

Furthermore, our correlational analyses revealed that Attention regulation was the only facet of the IAw, as assessed by the MAIA, to be significantly, albeit weakly, related to the participants' IAc, i.e., their ability to accurately perceive their bodily signals, as assessed by the performance in the HPT. This result is consistent with the fact that the Attention regulation scale assesses the participants' perception of their ability to actively direct and maintain attention on body signals, which is the very ability that was required to have a good performance in the HPT. This result is consistent with the report that Attention regulation was found to be the MAIA scale with the strongest correlation (Mehling et al., 2012) with the Private Body Consciousness subscale of the Body Consciousness Questionnaire (Miller et al., 1981), which specifically relates to the ability to notice bodily sensations such as the heart beating, thus involving IAc skills. Apart from this single, albeit interesting, significant result, the results of our analyses suggest the substantial independence of the IAc and IAw measures, supporting the notion that the assessment of IAc alone cannot provide a full comprehension of IAw. This independence is in line with more recent findings showing that subjective self-report measures of interoception ability, such as those provided by the MAIA, may diverge significantly from objective measures of IAc (Garfinkel et al., 2015). As a limitation of our IAc measures we need to note that three different measures of heart rate measures were used including manual palpation and chest auscultation that would potentially interfere with the participants' self-assessment. However, our analyses supported equivalence of the three methods. In addition, using exclusively self-report to assess IAw is a limitation that in future studies can—at least partially—be addressed following the method suggested by Garfinkel et al. (2015).

The complex pattern of correlations between the IAw, IAc, and ES revealed by our analyses would induce another important consideration supporting the above conclusion. While the correlational analyses showed that ES was not significantly related to the ability to accurately perceive bodily signals (IAc), multiple regression analyses showed that it was significantly and specifically related to different facets of IAw, particularly to the interaction between Trusting and Attention Regulation, which is the only MAIA scale to be related to the IAc. This pattern of results appears to provide additional support against the interoceptive sensitivity hypothesis (Ehlers and Breuer, 1992), which equates the IAw and IAc constructs, and, again, in favor of a conceptual distinction between these two constructs, as proposed by Ceunen et al. (2013) and Garfinkel et al. (2015). Moreover, the lack of a significant relation between IAc and ES would suggest that this latter construct does not simply correspond to the subjective experience of the intensity of emotional states. It has been shown that individuals with high IAc experience emotions as more intense (e.g., Wiens et al., 2000) and report them with a greater emphasis on the arousal dimension (Barrett et al., 2004). Based on these findings, we hypothesized that individuals with high IAc may be more emotionally susceptible, as they would tend to have greater arousal/excitement, especially “in situations, real, or imagined, of danger, offense, threat, or attack.” Contrary to our hypothesis, our finding indicates that this presumably greater activation in response to emotional stimuli does not automatically lead to the “feelings of discomfort, helplessness, inadequacy, and vulnerability,” and the “state of negative affect and a tendency to place oneself in a defensive position” that characterize individuals with high ES, as these feelings/emotions triggered by highly arousing situations are critically “due to the inability [of the individual] to control” this arousal/excitement (Caprara et al., 1983). This explanation is supported by reports (e.g., Füstös et al., 2013) that, although individuals with high IAc experienced negative emotions as more arousing, they were at the same time better able to actively down-regulate affect-related states, suggesting that the accurate perception of bodily states may be essential for adequate emotion regulation.

Alternatively, it has also been shown (Van't Wout et al., 2013) that IAc is not related to self-reported levels of the habitual use of emotion regulatory strategies, as assessed by the Emotion Regulation Questionnaire (ERQ: Gross and John, 2003). Therefore, it is possible that a positive association between IAc and the subjective experience of the intensity of emotions, combined with no association between IAc and emotion regulation ability canceled out any association between IAc and ES. Regrettably, we did not check our participants' habitual use of emotion regulation strategies, and thus we cannot verify this possible interpretation. Taken together, these findings suggest that the relationship between IAc and ES/emotion regulation appears to be more complex than we initially thought, and further investigation is needed to shed light on this issue.

Support for the conceptual distinction of the IAw and IAc constructs also comes from the analysis of the pattern of relations that these two constructs have with anxiety-related traits, which resembles a sort of “double dissociation.” First, as showed by Mehling et al. (2012), different MAIA scales are negatively correlated with trait anxiety, a general stable tendency to respond with anxiety to perceived threats in the environment, and to a lesser degree with anxiety sensitivity, a dispositional trait characterized by the fear of feelings related to anxiety, based on the belief that these symptoms have physical, psychological, or social consequences (Reiss and McNally, 1985; Reiss, 1991). Conversely, the HPT shows the opposite pattern, with stronger positive correlations with the anxiety sensitivity and weaker correlations with the trait anxiety (Domschke et al., 2010), suggesting that individuals with high anxiety sensitivity are generally more accurate in detecting their own heartbeat. Second, an increased IAc has been proposed as a risk factor for the development of state anxiety, trait anxiety or clinical disorders related to anxiety due to an attentional bias involving catastrophic misinterpretations of somatic signals (McNally, 2002; Schmidt et al., 2008; Perez Benitez et al., 2009; Domschke et al., 2010). Conversely, mindful meditation, and thus potentially improved IAw, is associated with the transition from (a) a form of conceptual and narrative thinking that is susceptible to evaluative interferences to (b) a form of experiential, non-evaluative, immediate sensing, in which the attention is focused directly on present-moment feelings and bodily sensations (Mehling et al., 2009; Daubenmier et al., 2013). These distinct modes of mind can thus determine whether body awareness is beneficial or maladaptive: “focusing attention directly on immediately experienced feelings appears to be adaptive, whereas an abstract ruminative self-focus appears to be maladaptive” (Mehling et al., 2012).

These distinct modes of minds correspond to distinct forms of self-awareness that are habitually integrated but, as shown with fMRI by Farb et al. (2007), “can be dissociated through attentional training: the self across time [i.e., the narrative focus] and the self in the present moment [i.e., the experiential focus].” Following 8 weeks of mindfulness meditation, an experiential focus was associated with three fMRI findings: (i) reduced activity of the medial prefrontal cortex, which supports narrative self-awareness by linking subjective experiences across time (Northoff and Bermpohl, 2004), providing narrative self-reference that preserves the identity stability across time (Gallagher, 2004); (ii) increased engagement of a right lateralized network (including the insula) for higher-order representations of the self (Critchley et al., 2004) through moment-to-moment self-reference and “experiential” focus, and (iii) decoupling of the functional connectivity between these two sets of areas. These findings are also in line with other studies investigating how meditation affects IAc and IAw measures. It has been shown that meditation, contemplative practice, or other mind–body interventions can longitudinally improve different facets of the IAw construct (e.g., Bornemann et al., 2014), but they seem to have no effect on the ability to accurately perceive bodily signals, as assessed by the HPT (Nielsen and Kaszniak, 2006; Khalsa et al., 2008; Melloni et al., 2013; Parkin et al., 2013).

To sum up, our analyses confirmed the factorial structure of the original MAIA in the new Italian version of the instrument. Moreover, they (a) confirmed the substantial independence of the IAc from self-reported IAw, (b) showed independence of IAc from ES measure, and (c) revealed a complex pattern of relationships between ES and distinct dimensions of IAw, such as Not-worrying, Emotional awareness, and the interplay between Attention regulation and Trusting. Taken together, these findings further highlight the need for a multidimensional assessment of the IAw construct and for a more in-depth analysis of its relationship with personality traits, such as ES or neuroticism.

### Conflict of interest statement

The authors declare that the research was conducted in the absence of any commercial or financial relationships that could be construed as a potential conflict of interest.

## Footnotes

^1^Note that when we applied the original structure of the MAIA the analyses yielded similar results, and thus are not reported here for the sake of brevity.
